# Utility of Asthma Control Questionnaire 7 in the assessment of asthma control*

**DOI:** 10.1590/S1806-37132014000200011

**Published:** 2014

**Authors:** Mariana Nadal Cardoso, Herberto José Chong, Lêda Maria Rabelo, Carlos Antônio Riedi, Nelson Augusto Rosário

**Affiliations:** Specialist in Allergy and Immunology. Federal University of Paraná School of Medicine Hospital de Clínicas, Curitiba, Brazil; Federal University of Paraná School of Medicine Hospital de Clínicas, Curitiba, Brazil; Federal University of Paraná School of Medicine Hospital de Clínicas, Curitiba, Brazil; Federal University of Paraná School of Medicine Hospital de Clínicas, Curitiba, Brazil; Federal University of Paraná School of Medicine Hospital de Clínicas, Curitiba, Brazil

**Keywords:** Asthma, Asthma, Questionnaires

## Abstract

Our objective was to evaluate the reproducibility of Asthma Control Questionnaire 7 (ACQ-7) in asthma patients, comparing our results against those obtained with the Global Initiative for Asthma (GINA) criteria. We evaluated 52 patients. Patients completed the ACQ-7, underwent spirometry, and were clinically assessed to determine the level of asthma control according to the GINA criteria, in two visits, 15 days apart. The ACQ-7 cutoff for uncontrolled asthma was a score of 1.5. The ACQ-7 showed good reproducibility, with a correlation coefficient of 0.73. The ACQ-7 identified a greater number of patients with uncontrolled asthma than did the GINA criteria; according to the GINA criteria, 47 patients (90.4%) presented with partially controlled asthma.

The objectives of asthma treatment are to control symptoms, prevent exacerbations, achieve the best possible lung function, allow patients to perform their regular activities, and prevent irreversible airway obstruction and death from asthma.^(^ ^1^ ^)^ Asthma control can be monitored in a variety of ways. Spirometry is a noninvasive technique to evaluate lung function in children who have asthma and are over 5 years of age. However, spirometry has limitations, which include the need for a professional trained in performing the test and the need for patient understanding and cooperation. Other noninvasive methods for monitoring asthma include measurement of peak expiratory flow, measurement of exhaled nitric oxide, and sputum examination for inflammatory cells.^(^ ^2^ ^)^ Asthma control questionnaires and quality of life questionnaires can be used in order to assess asthma control.^(^ ^1^ ^)^

In clinical practice, incorrect assessment of asthma control can result in inappropriate treatment. Therefore, efforts to provide physicians and patients with simple, rapid, and inexpensive instruments for accurate assessment of symptom control are warranted. The ideal tool should have good reproducibility and responsiveness, should provide cutoffs for uncontrolled asthma, should be practical, and should not pose health risks.^(^ ^3^ ^)^ Asthma control questionnaires are therefore important for the evaluation of disease control.

There are currently 17 previously validated questionnaires, all of which include questions regarding nocturnal symptoms and sleep disturbances; most assess the frequency of symptoms, the use of short-acting β_2_ agonists, and how asthma symptoms affect the performance of activities of daily living and physical exercise.^(^ ^3^ ^)^

One useful instrument is the Asthma Control Questionnaire (ACQ), which can be administered to asthma patients who are 12 years of age or older; the Spanish version and, more recently, the Brazilian Portuguese version of the ACQ have been validated for use.^(^ ^4^ ^)^ However, the reproducibility and responsiveness of ACQ-7, which includes six questions and one lung function parameter, have yet to be evaluated in Brazil.

The objectives of the present study were to evaluate the reproducibility of ACQ-7 and to compare ACQ-7 with the Global Initiative for Asthma (GINA) criteria in terms of their utility in identifying controlled and uncontrolled asthma.

The inclusion criteria were as follows: being 12 years of age or older; being under follow-up at one of the specialized clinics of the Federal University of Paraná School of Medicine *Hospital de Clínicas*, located in the city of Curitiba, Brazil; having been diagnosed with asthma and having received a diagnosis of asthma severity in accordance with the GINA criteria^(^ ^5^ ^)^; having received treatment with 800 µg/day of inhaled beclomethasone or equivalent, with or without long-acting β_2_ agonists, in the last six months. The exclusion criteria were as follows: need for systemic corticosteroids in the last three months; history of smoking in the last three months; current pregnancy; and presence of severe comorbidities.

Patients were assessed in two visits, the second occurring 15 days after the first. In the two visits, patients completed ACQ-7 and were evaluated by a specialist, who determined the level of asthma control on the basis of the GINA criteria.^(^ ^5^ ^)^

Patients completed the Brazilian Portuguese version of ACQ-7, which had previously been validated. The ACQ-7 cutoff for controlled asthma was a score = 0.75, and the ACQ-7 cutoff for uncontrolled asthma was a score = 1.5.^(^ ^6^ ^)^

Spirometry was performed with a portable spirometer (Microlab; Micro Medical Ltd., Rochester, UK), the Spida 5 software (Micro Medical Ltd.) and previously established reference values being used.^(^ ^7^ ^)^ In the two visits, spirometry was performed by the same professional, who was trained and qualified to do so. Values of FEV_1_ = 80% were considered normal.^(^ ^8^ ^)^ The level of asthma control was determined by a specialist, on the basis of the GINA criteria. However, because the specialist had no access to the spirometry results or ACQ-7 scores during the consultation, FEV_1_ was not taken into consideration.

Categorical variables are presented as frequency distributions, and continuous variables are presented as the mean percentage of absolute values. Statistical analysis was performed with the GraphPad Prism software (GraphPad Software Inc., San Diego, CA, USA). Pearson's correlation test was used in order to determine the ACQ-7 interclass correlation coefficient, the Wilcoxon test was used in order to determine the differences in FEV_1_ between the two visits, and the chi-square test was used in order to compare the variables. A convenience sample was used.

The study was approved by the Human Research Ethics Committee of the Federal University of Paraná *Hospital de Clínicas*. All patients gave written informed assent, consent, or both.

A total of 52 patients were included in the present study. The median age was 16.5 years (range, 12-84 years), and 65% of the patients were female. The mean height was 160.3 ± 7.5 cm, and the mean body mass index was 25.5 ± 6.3 kg/m^2^. Regarding asthma severity before treatment initiation, half of the patients were classified as having mild persistent asthma and half were classified as having moderate persistent asthma.

In order to evaluate the reproducibility of ACQ-7, we used the interclass correlation test for the ACQ-7 scores obtained in the initial visit and those obtained 15 days later (Figure 1).

**Figure 1:**
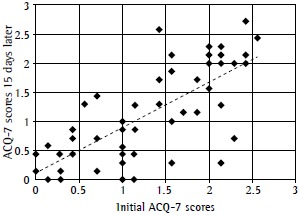
Correlation between the Asthma Control Questionnaire 7 (ACQ-7) scores obtained in the initial visit and those obtained 15 days later in the 52 patients studied. r = 0.73; 95% CI: 0.58-0.83; p < 0.0001.

A correlation coefficient of 0.73 was found, showing that the ACQ-7 scores obtained in the two visits correlated well. In order to determine whether there were any differences between asthma severity as assessed in the first visit and asthma severity as assessed in the second visit, we evaluated the variable FEV_1_ in isolation. No significant differences were found (p = 0.15).

We found no correlation between ACQ-7 scores and the level of asthma control by the GINA criteria (Table 1).

**Table 1:** Identification of patients with controlled asthma on the basis of the Global Initiative for Asthma criteria and Asthma Control Questionnaire 7 scores.

ACQ-7 score^a^	GINA criteria		
	Controlled asthma	Partially controlled asthma	Uncontrolled asthma
< 1.5	3	25	1
= 1.5	0	22	1

**ACQ-7:** : Asthma Control Questionnaire 7

GINA : Global Initiative for Asthma

a Cutoff for uncontrolled asthma = 1.5

* p = 0.06, chi-square test

Although 23 patients had ACQ-7 scores = 1.5 (i.e., uncontrolled asthma), only 2 were classified as having uncontrolled asthma on the basis of the GINA criteria. Of the 47 patients with partially controlled asthma by the GINA criteria, 22 were classified as having uncontrolled asthma on the basis of their ACQ-7 scores (i.e., = 1.5).

We found no correlation between FEV_1_ and the level of asthma control as determined by the GINA criteria. Most (90.3%) of the 52 patients included in the study were classified as having partially controlled asthma on the basis of the GINA criteria. Of those, 21 (45%) had FEV_1_ > 80% and 26 (55%) had FEV_1_ < 80%.

Of the 17 previously validated asthma control and quality of life questionnaires for monitoring symptoms in asthma patients, only 2 include items on lung function parameters: ACQ-7 and the Asthma Control Scoring System.^(^ ^3^ ^)^ The confirmation of the reproducibility of the Brazilian Portuguese version of ACQ-7 provides an instrument that includes items covering subjective symptoms and one lung function parameter and that can be used in clinical practice and research, its validity having been confirmed.^(^ ^4^ ^)^

We found no significant differences between the ACQ-7 scores obtained in the first visit and those obtained in the second. Because FEV_1_ values were similar between the two visits, ACQ-7 scores were expected to be similar as well. The fact that they were demonstrates the good reproducibility of ACQ-7.

In the present study, the level of asthma control as determined by ACQ-7 scores differed from the level of asthma control as determined by the GINA criteria. We found that ACQ-7 was more effective in identifying patients with uncontrolled asthma. These data show that the use of an instrument that includes items covering clinical symptoms and lung function parameters on an objective point scale can provide information to facilitate the clinical management of patients, given that asthma treatment progression is based primarily on the level of asthma control.

In a study similar to ours, the GINA criteria were compared with the Asthma Control Test (ACT), which is a 5-item questionnaire that does not include items on lung function parameters. It was concluded that ACT scores = 19 were useful in identifying patients classified as having uncontrolled or partially controlled asthma on the basis of the GINA criteria.^(^ ^9^ ^)^ Although the ACT does not include items on lung function parameters, the ACT cutoff for uncontrolled asthma correlates well with the ACQ.^(^ ^10^ ^)^ Therefore, the GINA criteria were expected to correlate well with ACQ scores. However, we found no such correlation in the present study.

We found that some of the patients who were classified as having partially controlled asthma on the basis of the GINA criteria had normal FEV_1_, whereas others had reduced FEV_1_. This finding suggests that the definition of partially controlled asthma does not accurately reflect lung function. When a patient is classified as having partially controlled asthma on the basis of the GINA criteria, the significance of this classification should be questioned, given that the patient might or might not have normal pulmonary function test results. In such patients, FEV_1_ should be measured in order to aid in making treatment decisions, given that it provides complementary information and is weakly associated with symptoms.^(^ ^11^ ^)^

The present study has some limitations. First, the ACQ-7 cutoffs aid in distinguishing between controlled and uncontrolled asthma; that is, they do not aid in assessing partially controlled asthma. Second, the GINA criteria and the National Asthma Education and Prevention Program lack a clear definition of asthma control.^(^ ^12^ ^)^

In conclusion, ACQ-7 showed good reproducibility in the present study. In addition, in the patients over 12 years of age in our sample, the level of asthma control by the GINA criteria differed from the level of asthma control as assessed by ACQ-7 scores, and ACQ-7 identified a greater number of patients with uncontrolled asthma than did the GINA criteria.
