# Supplementary material for: Utility of Asthma Control Questionnaire 7 in the assessment of asthma control
Source: J Bras Pneumol. 2014 Mar-Apr;40(2):171–4. doi: 10.1590/S1806-37132014000200011 (PMC4083642; doi:10.1590/S1806-37132014000200011)
Supplement: Translation

# Translation: Utilidade do instrumento *Asthma Control Questionnaire *7 na avaliação do controle da asma*

**Authors:** Mariana Nadal Cardoso, Herberto José Chong, Lêda Maria Rabelo, Carlos Antônio Riedi, Nelson Augusto Rosário

**Affiliations:** Hospital de Clínicas, Faculdade de Medicina, Universidade Federal do Paraná, Curitiba (PR) Brasil; Hospital de Clínicas, Faculdade de Medicina, Universidade Federal do Paraná, Curitiba (PR) Brasil; Hospital de Clínicas, Faculdade de Medicina, Universidade Federal do Paraná, Curitiba (PR) Brasil; Hospital de Clínicas, Faculdade de Medicina, Universidade Federal do Paraná, Curitiba (PR) Brasil; Hospital de Clínicas, Faculdade de Medicina, Universidade Federal do Paraná, Curitiba (PR) Brasil

**Keywords:** Asma/prevenção e controle, Asma/classificação, Questionários

## Abstract

Nosso objetivo foi avaliar a reprodutibilidade do *Asthma Control Questionnaire 7* (ACQ-7) em asmáticos e comparar os resultados com os critérios de controle da *Global Initiative for Asthma* (GINA). Foram avaliados 52 pacientes em duas visitas com intervalo de 15 dias entre si. Os pacientes responderam o ACQ-7, realizaram espirometria e foram avaliados clinicamente para verificar o controle da asma de acordo com a GINA nas duas visitas. Em relação ao ACQ-7, o ponto de corte para asma não controlada foi definido em 1,5. Os resultados de ACQ-7 demonstraram boa reprodutibilidade, com coeficiente de correlação de 0,73. O ACQ-7 identificou um maior número de pacientes com asma não controlada em relação aos critérios da GINA; segundo os critérios GINA, 47 pacientes (90,4%) tinham asma parcialmente controlada.

Os objetivos do tratamento da asma são manter o controle dos sintomas, prevenir exacerbações, manter a melhor função pulmonar possível, permitir atividades normais e evitar obstrução irreversível das vias aéreas e morte pela doença.^(^ ^1^ ^)^ Existem diversas ferramentas disponíveis para monitorar o controle da doença. A espirometria é uma técnica não invasiva para a avaliação da função pulmonar, utilizada em crianças maiores de 5 anos; porém, tem como limitação a necessidade de um profissional treinado para a realização do exame, além de exigir entendimento e cooperação do indivíduo. Outras ferramentas não invasivas para o monitoramento da asma são a medida de pico de fluxo expiratório, a do óxido nítrico exalado e a pesquisa de células inflamatórias no escarro.^(^ ^2^ ^)^ Para avaliar o controle da asma também é possível utilizar questionários de controle e de qualidade de vida.^(^ ^1^ ^)^

Na prática clínica, a avaliação do controle da asma pode resultar erroneamente em tratamento inadequado, o que justifica esforços para oferecer aos médicos e pacientes instrumentos simples, rápidos e de baixo custo para avaliar com precisão o controle dos sintomas. Uma ferramenta ideal para esse propósito deve ter boa reprodutibilidade e responsividade, deve fornecer valores de corte para identificar asma sem controle, ser prática e não oferecer riscos à saúde.^(^ ^3^ ^)^ Considerando essas características, os questionários para o monitoramento da asma são ferramentas importantes para avaliação do controle da doença.

Existem 17 questionários já validados e todos incluem perguntas sobre sintomas noturnos e interferência com o sono; a maioria avalia a frequência dos sintomas, o uso de β_2_-agonistas de curta ação e a interferência dos sintomas com a atividade diária e os exercícios físicos.^(^ ^3^ ^)^

Um instrumento útil é o *Asthma Control Questionnaire *(ACQ), que pode ser aplicado para asmáticos com idade = 12 anos, validado para uso em espanhol e, recentemente, para a língua portuguesa.^(^ ^4^ ^)^ No entanto, em nossa população, ainda não foram avaliadas a reprodutibilidade e a responsividade da sua versão 7 (ACQ-7), que inclui, além de seis perguntas, a prova de função pulmonar.

Os objetivos do presente estudo foram avaliar a reprodutibilidade do ACQ-7 e verificar a capacidade do ACQ-7 em identificar asma controlada e asma não controlada em comparação com os critérios definidos pela *Global Initiative for Asthma* (GINA).

Foram incluídos pacientes com idade = 12 anos, em acompanhamento nos serviços especializados do Hospital de Clínicas da Universidade Federal do Paraná, em Curitiba (PR), com diagnóstico de asma e de gravidade baseado nos critérios da GINA,^(^ ^5^ ^)^ em uso de beclometasona inalatória 800 µg/dia ou equivalente, associada ou não a β_2_-agonistas de longa ação nos últimos seis meses. Foram excluídos aqueles que necessitaram corticosteroides sistêmicos nos últimos três meses, história de tabagismo nos últimos três meses, gravidez atual e presença de comorbidades graves.

Os pacientes foram avaliados em duas visitas: a visita inicial e outra após 15 dias. Nas duas visitas, os pacientes responderam ao ACQ-7 e foram avaliados por um especialista com o objetivo de identificar o controle da asma baseado nos critérios da GINA.^(^ ^5^ ^)^

O ACQ-7 foi aplicado na sua versão em português, já validado para uso nessa língua. Foi definido como valor de corte para asma controlada um escore final = 0,75 e, para asma não controlada, valores = 1,5.^(^ ^6^ ^)^

A espirometria foi realizada com espirômetro portátil Microlab (Micro Medical Ltd., Rochester, Reino Unido); foram utilizados o software Spida 5 (Micro Medical Ltd.) e valores de referência estabelecidos.^(^ ^7^ ^)^ O exame foi realizado em ambas as visitas pelo mesmo profissional treinado e capacitado para obter os resultados de espirometria. Valores de VEF_1_ = 80% foram considerados normais. ^(^ ^8^ ^)^ Na consulta com um especialista, foi definido o controle de asma baseado nos critérios da GINA, excluindo-se o VEF_1_, pois, durante a consulta, o profissional não teve acesso aos resultados da espirometria nem ao escore final de ACQ-7.

As variáveis categóricas foram apresentadas em distribuição de frequências, e as variáveis contínuas foram apresentadas em média da porcentagem do valor absoluto. A análise estatística foi realizada com o software GraphPad Prism (GraphPad Software Inc., San Diego, CA, EUA). Foram aplicados os testes de correlação de Pearson para a correlação interclasse do ACQ-7, o teste de Wilcoxon para verificar diferenças no VEF_1_ entre as visitas e o teste do qui-quadrado para a comparação das variáveis entre si. Foi utilizada uma amostra de conveniência.

O estudo foi aprovado pelo Comitê de Ética em Pesquisa em Seres Humanos do Hospital de Clínicas da Universidade Federal do Paraná. Os pacientes assinaram termo de assentimento e/ou termo de consentimento livre e esclarecido.

Foram incluídos 52 pacientes, com mediana de idade de 16,5 anos, com variação de 12 a 84 anos, sendo 65% do gênero feminino. A média de estatura foi de 160,3 ± 7,5 cm e a média de índice de massa corpórea foi de 25,5 ± 6,3 kg/m^2^. Quanto à gravidade da asma antes do início do tratamento, metade dos pacientes foi classificada como com asma leve persistente e o restante como asma moderada persistente.

Para avaliar a reprodutibilidade do ACQ-7, foi aplicado o teste de correlação interclasse para os valores de ACQ-7 da visita inicial e da visita após 15 dias (Figura 1).

**Figura 1:** Correlação dos resultados do Asthma Control Questionnaire 7 (ACQ-7) na visita inicial e na visita 15 dias depois nos 52 pacientes estudados. (r = 0,73; IC95%: 0,58-0,83; p < 0,0001)

Foi encontrado um índice de correlação de 0,73, demonstrando que os valores de ACQ-7 nas duas visitas tiveram boa correlação entre si. Foi avaliada de forma isolada a variável VEF_1_ para verificar diferenças de gravidade da asma nas duas visitas, e não houve diferença estatística (p = 0,15).

Comparando os valores de ACQ-7 com a identificação de controle da asma pelos critérios da GINA, não foi possível encontrar uma correlação entre os dois métodos (Tabela 1).

**Tabela 1:** Identificação dos pacientes com asma controlada utilizando critérios da Global Initiative for Asthma e o Asthma Control Questionnaire 7.

ACQ-7^a^	Critérios da GINA na definição de asma		
	Controlada	Parcialmente controlada	Não controlada
< 1,5	3	25	1
= 1,5	0	22	1

**ACQ-7:** : *Asthma Control Questionnaire 7*

GINA : *Global Initiative for Asthma*

a Ponto de corte para a classificação de asma não controlada = 1,5

* p = 0.06, teste do qui-quadrado

Enquanto 23 pacientes tiveram escore final de ACQ-7 = 1,5, definindo ausência de controle, apenas 2 foram identificados como portadores de asma não controlada pelos critérios da GINA. Dos 47 pacientes com asma parcialmente controlada de acordo com os critérios da GINA, 22 foram definidos pelo ACQ-7 como portadores de asma não controlada, com escore final = 1,5.

Quando comparados os valores de VEF_1_ com o controle da asma definido pela GINA, não foi observada uma correlação dos valores de VEF_1_ com o controle de asma. Entre os 52 pacientes incluídos no estudo, a grande maioria (90,3%) foi definida como com asma parcialmente controlada de acordo com os critérios da GINA, e desses, 21 (45%) apresentaram VEF_1_ > 80% e 26 (55%), VEF_1_ < 80%.

Entre os 17 questionários de controle e qualidade de vida disponíveis e validados para o monitoramento dos sintomas em pacientes asmáticos, apenas 2 questionários incluem parâmetros de função pulmonar: ACQ-7 e *Asthma Control Scoring System*.^(^ ^3^ ^)^ A comprovação da reprodutibilidade do ACQ-7 em seu uso na língua portuguesa fornece uma ferramenta que inclui sintomas subjetivos e um parâmetro de função pulmonar, possível de ser utilizada na prática do médico e em pesquisa clínica com validação comprovada.^(^ ^4^ ^)^

Quando foram avaliados os valores de ACQ-7 nas duas visitas, observou-se que não houve alterações significativas. Como nas duas visitas os valores de VEF_1 _foram semelhantes, era esperado que os valores de ACQ-7 também não sofressem alterações, demonstrando que, em situações clínicas semelhantes, o ACQ-7 teve boa reprodutibilidade.

No presente estudo, o ACQ-7 e os critérios da GINA não identificaram de forma semelhante o grau de controle da asma. Foi possível observar que o ACQ-7 teve maior capacidade de identificar os pacientes com asma não controlada. Esses dados mostram que a utilização de uma ferramenta que inclua em sua avaliação sintomas clínicos e prova de função pulmonar, colocando-os em escala de pontos de uma forma objetiva, pode nos fornecer informações que facilitem o manejo clínico do doente, já que a progressão no tratamento será baseada principalmente no controle ou não da asma.

Em um estudo semelhante, foram comparados os critérios da GINA e o *Asthma Control Test *(ACT), questionário com 5 itens, mas que não inclui prova de função pulmonar. Concluiu-se que valores de ACT = 19 foram úteis para identificar pacientes com asma não controlada e parcialmente controlada de acordo com os critérios da GINA.^(^ ^9^ ^)^ Apesar de o ACT não incluir prova de função pulmonar, quando comparado com o ACQ, seu valor de corte para a identificação de asma não controlada tem boa correlação.^(^ ^10^ ^)^ Portanto, seria esperado também que houvesse uma boa correlação entre os critérios da GINA e ACQ, o que não foi encontrado no presente estudo.

Foi observado que parte dos pacientes definidos como portadores de asma parcialmente controlada pelos critérios da GINA apresentavam valores normais de VEF_1_ enquanto outra parte apresentava VEF_1_ reduzido, o que sugere que a definição de asma parcialmente controlada não reflete de maneira adequada a função pulmonar. Quando definido um paciente com asma parcialmente controlada utilizando os critérios da GINA, deve-se questionar o seu significado, já que esse pode ter prova de função pulmonar normal ou não. O valor de VEF_1_ deveria ser definido nesse grupo de pacientes para auxiliar na decisão do tratamento, uma vez que oferece informações complementares e possui baixa associação com sintomas.^(^ ^11^ ^)^

O presente estudo tem algumas limitações. Primeiro, os valores de corte do ACQ-7 diferenciam asma controlada de asma não controlada, ou seja, não avaliam asma parcialmente controlada. Segundo, não há definições estabelecidas para o controle da asma, nem pelos critérios da GINA, nem pelo *National Asthma Education and Prevention Program*.^(^ ^12^ ^)^

Em conclusão, o ACQ-7 teve boa reprodutibilidade no presente estudo. Além disso, a avaliação do controle da asma nos pacientes acima de 12 anos de nossa amostra não foi semelhante entre os métodos avaliados (ACQ-7 e GINA), e o ACQ-7 identificou um maior número de pacientes com asma não controlada do que em relação aos critérios da GINA.
